# Editorial: Navigating the landscape of FAIR data sharing and reuse: repositories, standards, and resources

**DOI:** 10.3389/fninf.2024.1387758

**Published:** 2024-03-01

**Authors:** Maaike M. H. van Swieten, Christian Haselgrove

**Affiliations:** ^1^Netherlands Comprehensive Cancer Organization (IKNL), Utrecht, Netherlands; ^2^UMass Chan Medical School, Worcester, MA, United States

**Keywords:** FAIR principles, neuroinformatics, data sharing, data reuse, repositories, standards

In response to the expanding landscape of neuroscience data and the diverse array of formats emerging from various research communities, the scientific community faces a pressing challenge in traditional data management, sharing, and mining methods. The push for data sharing mandates and the increasing demand for open data utilization (e.g., National Institutes of Health, 2023) have prompted the evolution of sophisticated methodologies and tools. These advancements aim to empower researchers in effectively exploring, mining, and integrating datasets. However, the growing number of resources in this rapidly evolving field poses a substantial hurdle for researchers attempting to navigate this complex landscape.

As the scientific community strives to uphold data sharing mandates and embrace open data principles, it becomes imperative to equip researchers with the awareness and knowledge necessary to navigate this landscape successfully. This Frontiers in Neuroinformatics Research Topic was designed to showcase exciting recent developments in the field and offer a nuanced overview of available resources, with a focus on ensuring that data are findable, accessible, interoperable, and reusable—adhering to the FAIR principles (Wilkinson et al., 2016).

The Research Topic reflects the broad extent of the FAIR landscape. While the FAIR principles apply directly to data, repositories, and standards such as ontologies, satisfying the full intent of the FAIR principles often requires more diverse considerations, as exemplified here: from atlases and software to workflows and even data governance.

A comprehensive overview of the components and practices required to achieve FAIR in neuroscience along with the perspectives on the past, present and future of a FAIR infrastructure for neuroscience, are provided in the review article by Martone. This article also compares large next-generation neuroscience infrastructures, including EBRAINS, CONP, SPARC, DANDI, Open Neuro, and BRAIN/Minds.

This Research Topic also features four articles about FAIR repositories, namely Brain-CODE for general neuroscience data, COINSTAC Vaults and Image and Data Archive (IDA) for neuroimaging data, and GAAIN, DPUK, ADDI for Alzheimer's and dementia-related data. Each article delves into the challenges and solutions related to making the repository FAIR, the governance and sovereignty concerns, and the steps taken to enhance the user experience. These repositories mainly vary in their technical implementations and mechanisms for managing data governance requirements and sovereignty, particularly for datasets containing sensitive or personal data, which require specific permissions.

COINSTAC, for example, addresses challenges through federated analysis, enabling researchers to analyze datasets without public data sharing (Martin et al.). The introduction of COINSTAC Vaults (CVs) enhances this capability by providing standardized, persistent datasets that seamlessly integrate with COINSTAC's federated analysis. CVs offer a user-friendly interface, promoting self-service analysis and filling a crucial gap in the data sharing ecosystem.

Other platforms like DPUK, GAAIN, and ADDI rely on two core design principles, such as “trust-by-design” and “data federation”, actively developing a range of innovative solutions to enhance large-scale data access (Toga et al.). This includes simplifying stakeholder involvement through streamlined data sharing agreements, introducing decentralized data sharing solutions, and establishing universally accessible analysis through workspaces and containerized software.

The IDA, run by the Laboratory of Neuro Imaging, presents an alternative approach to managing and reusing multi-center data (Neu et al.). Serving as a central hub for collaborative groups, it facilitates data transfers and offers a suite of informatics tools. These tools are designed to support in various tasks, including de-identifying, integrating, searching, visualizing, and sharing a diverse range of neuroscience data. Researchers maintain full control over the data stored in the IDA, benefiting from a reliable infrastructure that safeguards and preserves research data.

Brain-CODE, a large-scale neuroinformatics platform, supports the collection, storage, federation, sharing and analysis of different data types across different types of brain disorders. Behan et al. discuss the data sharing processes on Brain-CODE, aligning them with the FAIR principles. Brain-CODE not only provides extensive metadata for interactive searches and the ability to generate subsets of data, but also focuses on mechanisms and services that facilitate interoperability and the combination of data using advanced privacy preserving record linking and homomorphic encryption. Sensitive data can be accessed within a secure workspace on Brain-CODE, and public datasets can be exported to a locally device.

Currently, repositories predominantly address data governance concerning data derived from human subjects. However, there is a noticeable absence of regulatory frameworks for non-human data, despite divergent legal and ethical principles across countries about the generation of animal data. Eke et al. advocate for the establishment of animal data governance, proposing to delineate and collect metadata related to ethical considerations. This proposal aims to enhance data transparency and promote the FAIR principles within the context of animal research.

Despite the growing number of datasets on repositories mentioned above, a considerable amount of data remains underutilized and inaccessible, especially smaller-sized datasets. This is often attributed to the quality of the associated metadata and the degree of annotations. The NeuroBridge platform (Wang et al.) and the NeuroBridge Ontology (Sahoo et al.) offer innovative approaches for extracting metadata related to study design and data collection from full-text papers through ontology developments and machine-learning-based natural-language processing. By harnessing the search capabilities of the NeuroBridge platform, researchers can pinpoint neuroimaging datasets tailored to their specific research questions, thereby promoting data reuse.

Queder et al. propose an alternative method for standardizing and annotating neuroimaging datasets. Neuroimaging datasets are typically organized with the Brain Imaging Data Structure (BIDS) (Gorgolewski et al., 2015), which, while useful for file-naming and controlling directory structures, does not support querying across datasets. To address this, Queder et al. introduce NIDM-Terms, a formal set of user-friendly terminology management tools, and associated software to annotate BIDS datasets with a Neuroimaging Data Model (NIDM) semantic web representation.

Standardization of metadata is not only crucial for neuroimaging data, but also for anatomical studies that heavily rely on brain atlases. Kleven et al. provide a guide on the interpretation, navigation, spatial registration, data visualization, and transparent reporting of findings using different types of murine brain atlases. In addition, Blixhavn et al. provide a workflow defining the anatomical location of data elements in rodent brains as geometric objects based on atlas coordinates, which can be stored in a standardized file format. Using this method, disparate multimodal and multilevel neuroscience data can be co-visualized in three-dimensional digital brain atlases, enabling spatial data queries.

Even when data are shared, data accessibility, interoperability and reusability can be hindered by the use of proprietary data formats, especially when accompanying software becomes unavailable or unsupported. For proprietary electrophysiological data recorded with the DAPSYS software, Konradi et al. designed PyDapsys to enable direct opening of recorded files in Python and save them as NIX files, commonly used for open research in electrophysiology. This software promotes transparency and long-term accessibility in neuroscience research.

In this Research Topic, researchers describe various challenges and solutions surrounding FAIR data sharing and reuse in neuroscience. Their insights cover best practices for achieving data interoperability, the development of tools supporting scientists in data management and annotation, and the formulation of workflows to enhance the value of current and future data. We anticipate that the repositories, standards, and resources discussed in this Research Topic will not only simplify data sharing but also elevate reproducibility and foster widespread reuse of valuable neuroscience data. This collective effort holds the potential to significantly advance collaborative neuroscientific research.

## Author contributions

MvS: Writing—original draft, Writing—review & editing. CH: Writing—review & editing.
